# Apparent density, trypanosome infection rates and host preference of tsetse flies in the sleeping sickness endemic focus of northwestern Uganda

**DOI:** 10.1186/s12917-021-03071-w

**Published:** 2021-11-29

**Authors:** Robert Opiro, Robert Opoke, Harriet Angwech, Esther Nakafu, Francis A. Oloya, Geoffrey Openy, Moses Njahira, Mercy Macharia, Richard Echodu, Geoffrey M. Malinga, Elizabeth A. Opiyo

**Affiliations:** 1 Department of Biology, Faculty of Science, Gulu University, P.O Box 166, Gulu, Uganda; 2 Department of Biology, Faculty of Science, Muni University, P.O Box 725, Arua, Uganda; 3 Department of Molecular Biology, College of Veterinary Medicine, Animal Resources and Biosecurity, Makerere University, P.O Box 7062, Kampala, Uganda; 4 Department of Biosystems Engineering, Faculty of Agriculture and Environment, Gulu University, P. O Box 166, Gulu, Uganda; 5 Biosciences Eastern and Central Africa-International Livestock Research Institute Hub, P. O Box 30709, Nairobi, Kenya; 6 Department of Forestry, Biodiversity and Tourism, Makerere University, PO Box 7062, Kampala, Uganda

**Keywords:** CO1, Cytb, Nested PCR, Reservoir hosts, Mixed infection, Adjumani, Moyo

## Abstract

**Background:**

African trypanosomiasis, caused by protozoa of the genus *Trypanosoma* and transmitted by the tsetse fly, is a serious parasitic disease of humans and animals. Reliable data on the vector distribution, feeding preference and the trypanosome species they carry is pertinent to planning sustainable control strategies.

**Methodology:**

We deployed 109 biconical traps in 10 villages in two districts of northwestern Uganda to obtain information on the apparent density, trypanosome infection status and blood meal sources of tsetse flies. A subset (272) of the collected samples was analyzed for detection of trypanosomes species and sub-species using a nested PCR protocol based on primers amplifying the Internal Transcribed Spacer (ITS) region of ribosomal DNA. 34 blood-engorged adult tsetse midguts were analyzed for blood meal sources by sequencing of the mitochondrial cytochrome c oxidase 1 (COI) and cytochrome b (cytb) genes.

**Results:**

We captured a total of 622 *Glossina fuscipes fuscipes* tsetse flies (269 males and 353 females) in the two districts with apparent density (AD) ranging from 0.6 to 3.7 flies/trap/day (FTD). 10.7% (29/272) of the flies were infected with one or more trypanosome species. Infection rate was not significantly associated with district of origin (Generalized linear model (GLM), χ^2^ = 0.018, *P* = 0.895, df = 1, *n* = 272) and sex of the fly (χ2 = 1.723, *P* = 0.189, df = 1, *n* = 272). However, trypanosome infection was highly significantly associated with the fly’s age based on wing fray category (χ^2^ = 22.374, *P* < 0.001, df = 1, n = 272), being higher among the very old than the young tsetse. Nested PCR revealed several species of trypanosomes: *T. vivax* (6.62%), *T. congolense* (2.57%), *T. brucei* and *T. simiae* each at 0.73%. Blood meal analyses revealed five principal vertebrate hosts, namely, cattle (*Bos taurus*), humans (*Homo sapiens*)*,* Nile monitor lizard (*Varanus niloticus*), African mud turtle (*Pelusios chapini*) and the African Savanna elephant (*Loxodonta africana*).

**Conclusion:**

We found an infection rate of 10.8% in the tsetse sampled, with all infections attributed to trypanosome species that are causative agents for AAT. However, more verification of this finding using large-scale passive and active screening of human and tsetse samples should be done. Cattle and humans appear to be the most important tsetse hosts in the region and should be considered in the design of control interventions.

## Background

African trypanosomes, members of the genus *Trypanosoma*, are flagellated parasitic protozoa that cause diseases generally known as African trypanosomiasis. The diseases are known as sleeping sickness (or human African trypanosomiasis, HAT) and Nagana (or animal African trypanosomiasis, AAT) and are inextricably linked in humans and livestock, respectively. HAT, though on a gradual decline, still threatens millions of people in 37 countries in sub-Saharan Africa; the disease causes severe morbidity and mortality, with over 70 million people at risk of being infected [1]. AAT on the other hand is a major constraint to livestock production causing massive economic burden in sub-Saharan Africa. The Food and Agricultural Organization of the United Nations (FAO) estimates that Africa loses up to US$1.5 billion annually as a result of the disease [2]. Thus, these diseases together, have both negative health and economic impacts.

In Uganda, vector-borne diseases, notably AAT presents a major constraint to livestock productivity [3, 4]. Poor livestock health as a result of AAT denies farmers draught power and manure thereby contributing to poverty and hunger in the tsetse-infested areas [5, 6]. Regarding HAT, despite the current small number of cases, Uganda is the only country where both the chronic (caused by *T. b. gambiense*) and acute (caused by *T. b. rhodesiense*) forms of the disease occur. The *T. b. gambiense* form occurs in the northwestern corner of the country (where this study was conducted) while *T. b. rhodesiense* is in the Eastern and Southern part of Uganda. Evidence already point to a danger of merger of the two HAT belts, fueled by animal movements [7, 8], and vector migration northwards [9–11]. This underscores the need for research geared towards providing information to support sustainable control in the country.

Northwestern Uganda has traditionally been known as an endemic sleeping sickness focus but just like in the rest of the continent, there has been a gradual decline in the number of new cases. For example, passive screening conducted in 2014 detected just nine cases in the region [12], and in 2018, out of the 10,000 individuals screened, none was infected with *T*. *b*. *gambiense* [13]. In the same year, a tsetse control intervention was expanded to cover the main *gambiense* HAT foci in West Nile to curtail transmission of *gambiense,* and this reduced the prevalence to below the elimination threshold (1 new case per 10,000 population), making elimination as a public health problem on course across this study area if status is maintained [13]. Regarding AAT, there are numerous confirmed and anecdotal reports of the presence of the disease among cattle keeping households. Moreover, a recent study [14] found the prevalence of *T*. *b. brucei* in local cattle, pigs and tsetse flies as follows: 1.9, 6.3 and 1.8%, respectively. Therefore, AAT still remains a public health challenge in the region.

Despite the existence of various drugs effective against AAT, chemoprophylaxis and treatment are expensive and usually ineffective, moreover it is impossible to treat all wild and domestic hosts [15]. Furthermore, improper use of therapeutic drugs has led to the emergence of drug resistance in animal trypanosomes [16]. Vector control therefore offers a more viable strategy to disease management [16, 17] and it remains the only available strategy capable of protecting humans from acquiring infection. However, effective vector control requires reliable and accurate data on distribution, trypanosome infection status and preferred hosts of the vectors. The main objective of this study was to update our knowledge on the current risk of Human and Animal African trypanosomiasis in the endemic focus of Northwestern Uganda. The specific objectives were to; (i) assess the apparent density and tsetse trypanosome infection status, and (ii) investigate the preferred sources of blood meals of tsetse flies trapped in the West Nile districts of Adjumani and Moyo.

## Methods

### Study sites and sample collection

**Fig. 1:**
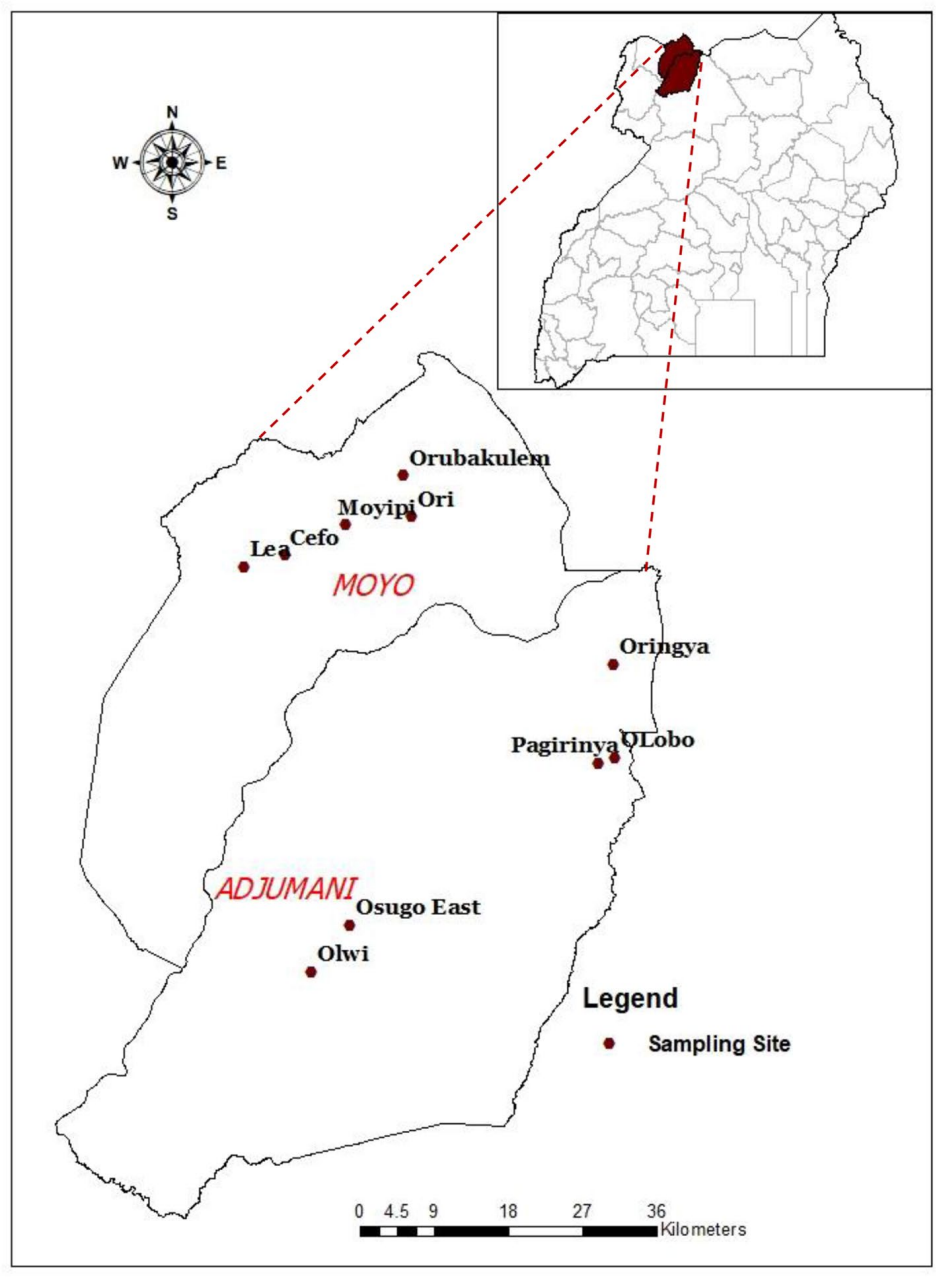
Map of study area showing sampled villages. Map was created by the authors using ArcGIS version 10.3.1

The study was carried out in Adjumani (3.3784° N, 31.7822° E) and Moyo districts (3.6527° N, 31.7281° E) in northwestern Uganda (Fig. 1). The region has rainfall periods that run from March–May and from July–November, with a short dry spell in June and a fairly long period of dryness from December to February. The vegetation is a mixture of forests and savannah, with open woodland, grassland, and shrubs. There are several fast-running streams passing through subsistence farms with low plains and rolling hills and valleys that slope towards the river Nile. The typical riverine habitats are suitable for *Glossina fuscipes fuscipes*, the principal tsetse vector in this area. The population is largely rural, practicing mixed crop and livestock farming, consisting of food and cash crops such as tobacco, and livestock, mainly cattle, goats, sheep and pigs.

**Fig. 2:**
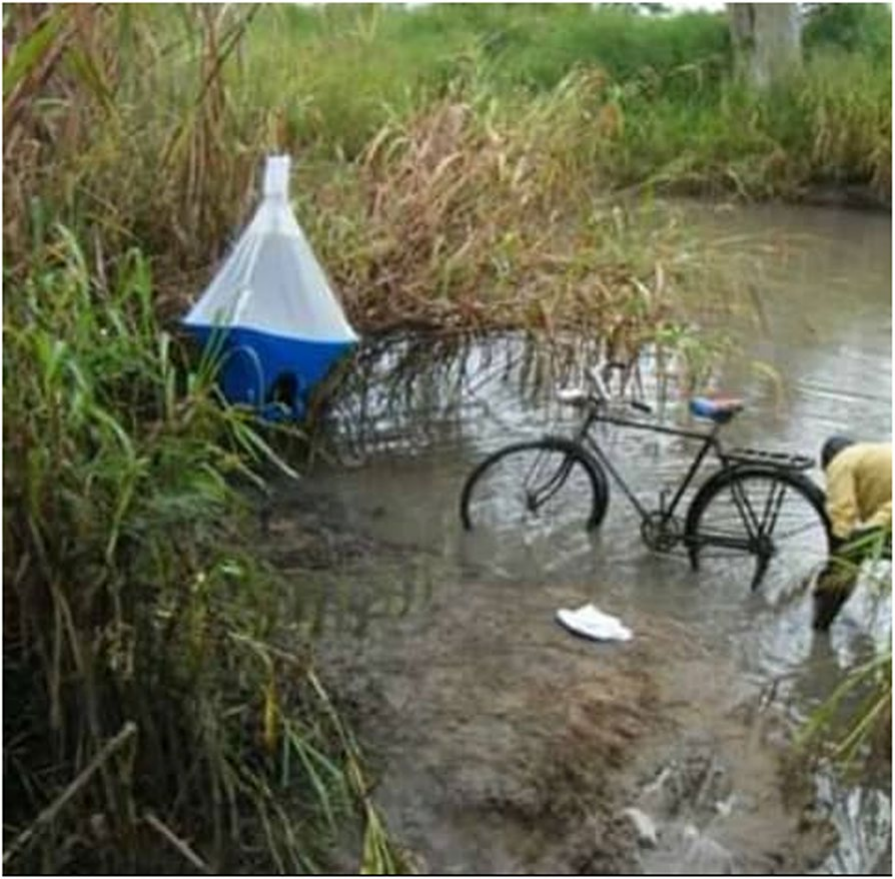
A biconical trap baited with cow urine and acetone to enhance trapping efficiency deployed along a river in the study area

Tsetse trapping was conducted using biconical traps [18] baited with acetone and cow urine, deployed along suitable habitats, targeting majorly areas of human and animal activities (Fig. 2). At each site, an average of 10 traps were mounted atleast 100 m apart in different vegetation types for three consecutive days and in well shaded areas to minimize fly mortality due to excessive heat. The traps were deployed in the villages of Olwi, Olobo, Oringya, Osugo East and Pagirinya in Adjumani district, and in Orubakulem, Ori, Lea, Cefo and Moyipi villages in Moyo district. The geographical coordinates of each trap was recorded using a handheld Global Positioning Systems (GPS) unit (GPS 12 XL, Garmin Ltd. 2003, Olathe, Kansas, USA). We also recorded the trap codes, sex of the captured flies and dates of collection. To prevent the attack of ants on the flies in the traps, each supporting pole was smeared with grease. Trapped tsetse flies were collected every 24 h for at least three consecutive days [19] . After each collection, tsetse flies were identified morphologically, counted and sorted into teneral and non-teneral as described by Laveissière et al. [20]. The tsetse flies were assigned to one of the six age categories, according to the degree of wear or fraying observed on the hind margin of the wing as described by Jackson [21]. After categorizing the wing fray (WF), the age of the fly was estimated using directions for estimating the mean age of a sample of tsetse flies as outlined in the FAO Training manual for tsetse control personnel [22]. The ages of tsetse flies based on wing fray categories were later pooled as “young tsetse” (WF1–2), “old tsetse” (WF 3–4) and “very old tsetse” (WF 5–6) for statistical analysis. Non-teneral flies were then preserved in 70% ethanol in sealed eppendorf tubes until required for subsequent DNA extraction and PCR assays.

### DNA extraction

The ethanol preserved tissues of each tsetse sample was placed together in a single tube and genomic DNA extracted using Purelink™ extraction kit from Invitrogen following the manufacturer’s instructions. The supernatant was used either directly for PCR or stored at − 20 °*C. prior* to their use or storage, DNA samples were electrophoresed in a 1% agarose gel in 0.5× TBE buffer at 100 V for 45 mins. The quality of DNA in the sample was then estimated by comparing florescent yield of the sample with standard cut Lambda DNA run alongside the DNA sample.

### Identification of different trypanosome species

To detect trypanosome DNA, we employed the nested PCR protocol described by Cox et al. [23], using the same primer sequences but with slight modifications in amplification conditions. The outer primer sequences were ITS1 (5-GAT TAC GTC CCT GCCATT TG-3) and ITS2 (5-TTG TTC GCT ATC GGTCTT CC-3), and inner primer sequences ITS3 (5-GGA AGC AAA AGT CGT AACAAG G-3) and ITS4 (5-TGT TTT CTT TTC CTCCGC TG-3). PCR amplifications were performed in two rounds. The first round was performed in a final reaction volume of 20 μL containing 10 pmol of each primer, the Bioneer*AccuPower®* PCR premix (Bioneer Corporation), and 2 μL of each DNA template. The amplification conditions began with 1 cycle of denaturation at 95 °C for 5 min followed by 40 amplification cycles at 94 °C for 1 min, 55 °C for 1 min, and 72 °C for 2 min. In the second round, 2 μL of the amplified product from the first round was placed in a fresh tube and 20 μL of the reaction mixture was added as described above for the outer primers, except that the outer primers (ITS1 and 2) were substituted with the inner primers (ITS3 and 4). The amplifications conditions were identical to the one described for the first PCR round. To minimize bias due to false positives during repeated PCRs, negative controls in which DNA templates were replaced with sterile distilled water as well as positive control DNAs (of each trypanosome species) were included in all PCR reactions. All reactions were carried out using a GeneAmp 9700 thermal cycler PCR system (Applied Biosystems). After the nested PCR, 5 μL of the amplified products were loaded on a casted 1.8% agarose gel, which was subsequently stained with a Gel Red nucleic acid stain, with a 75 bp gene marker. These were run in a Mupid®-exu Sub-marine electrophoresis gel tank (Helix Technologies Inc. MEXO 0800137) for 45 mins at 100 V in 0.5X TBE buffer. The gels were then visualized under ultra-violet illumination and photographed. Trypanosomes species and subspecies were identified by comparing the molecular sizes of their DNA fragments with the documented band sizes of trypanosome species [23] (Table 1). For *T. brucei*, further investigation was done by running a second PCR for diagnosis of *T. b. gambiense* employing a nested-PCR with a first reaction using TgsGP1/2 primers [24] and a second one with TgsGP sense2/antisense2 primers described by Morrison et al. [25].

**Table 1:** Expected and obtained band sizes for amplification using nested ITS primers (Adapted from Cox et al., [23])

Species	Expected band size (bp) from NCBI database	Band sizes (bp))
*T. congolense* (Forest)	1513	1501
*T. congolense* (Kilifi)	1422	1430
*T. congolense* (Savannah)	1413	1408
*T. congolense* (Tsavo)	954	951
*T. brucei*	1207–1224	1215
*T. simiae*	850	847
*T. vivax*	611	620
*T. theileri*	988	998

### Identification of tsetse blood meal sources

To test for the origin of blood meals, samples of DNA from residual blood meal in tsetse midguts were amplified using PCR with universal primers complementary to the conserved region of mitochondrial DNA (mtDNA) CO1 and cytb gene as detailed by Muturi et al. [26]. The primer sequences for the CO1 gene was VF1d_t1 (5′ TGTAAAACGACGGCCAGTTCTCAACCAACCACAARGAYATYGG- 3′) and VR1d_t1 (5′-CAGGAAACAGCTATGACTAGACTTCTGGGTGGCCRAARAAYCA- 3′) and for the Cyt b was (Cb5’-CCATCCAACATCTCAGCATGATGAAA-3′) and (Cb2 5′-CCCCTCAGAATGATATTTGTCCTCA-3′) as described by Ivanova et al. [27] and Kocher et al. [28], respectively. Both PCR reactions were performed in a total volume of 20 μl containing 10 pmol of each primer, 10 mMTris-Cl, pH 8.3 and 50 mMKCl, 1.5 mM MgCl_2_, 2.5 mMdNTPs, 2 μL of the DNA template and 1unit DreamTaq™ DNA polymerase (Fermentas Life Sciences). The PCR was then carried out in a GeneAmp PCR System 9700 (Applied Biosystems) thermocycler. The conditions for the CO1 PCR were as follows: initial denaturation at 94 °C for 5 min, followed by 45 cycles of denaturation at 94 °C for 30 s, annealing at 55 °C for 1 min, and primer extension at 72 °C for 30 s. For Cyt b PCR, conditions were: initial denaturation at 94 °C for 5 min, followed by 40 cycles of denaturation at 94 °C for 45 s, annealing at 55 °C for 45 s, and primer extension at 72 °C for 30 s. Positive and negative controls were included in each PCR assay. The positive controls were cattle genomic DNA obtained from the Animal Health Unit at ILRI Research Laboratories. Negative controls consisted of the Master Mix for each PCR and fresh Milli Q water obtained from the Central Core of the BeCA hub laboratories. To analyze the amplicons, 5 μL of the PCR product, as well as negative and positive controls, was resolved in a 1.8% agarose gel at 80 V for 45 min and visualised under UV transilluminator following gel red staining. The amplified products were visualized under ultra-violet illumination and a picture of the gel taken using the gel documentation and analysis systems mounted with a high-performance CCD Camera-COHU.

The PCR products were gel-purified using the GeneJet™ kit (catalog no K0702 EU) following the manufacturer’s instructions and submitted to the BeCA-ILRI Hub Sequencing Unit (Segolip) for sequencing using Bigdye™ Terminator. Sequencing was done bi-directionally using the inner amplification primers for the CO1 and *cyt b* genes. Consensus sequences were then generated by contiguation functions of the CLC Main Workbench Version 6.6.2 and manually edited, where necessary, by reference to the chromatograms. Vertebrate species were confirmed by sequence alignments with those already deposited in GenBank database using the Basic Local Alignment Search Tool (BLAST, https://blast.ncbi.nlm.nih.gov/Blast.cgi) [29]. Sequences of a given pair-wise alignment from positive PCR products with high percentage similarity (identity matches of 90–98%) and lowest E- values were selected as the most likely species of host.

### Data analyses

The average Apparent density (AD), expressed as the average number of flies caught per trap per day (flies/ trap/ day or FTD), was calculated to obtain the data on tsetse distribution in the area for each trapping site using the formula: FTD = ΣF/T × D, where, ΣF is the total number of tsetse flies caught, T is the number of traps deployed and D is the number of days of trapping in the field [30]. Tsetse infection rate was calculated by dividing the number of flies infected with trypanosomes by the total number of flies analysed, and expressed as percentages. To determine the predictors of tsetse infection, we fitted a generalized linear model (GLM) with a negative binomial error distribution and a log link function (prevalence of infection as the response variable and the fly’s sex, district of origin and age based on wing fray category as covariates). Values of *p*-value < 0.05 were considered significant at 95% confidence interval (CI). We tested whether the average number of flies caught per trap per day differed between the two districts under study using a non-parametric Mann-Whitney U test. All statistical tests were performed using SPSS software (version 21.0.1, SPSS Inc., Chicago, IL, USA).

## Results

### Entomological survey

We caught 622 tsetse flies (269 males and 353 females) from 109 traps deployed in 10 villages (five in each district) (Table 2). Of these, 320 flies were caught from Adjumani and 302 from Moyo districts, respectively. All the tsetse flies caught belonged to the *Glossina fuscipes fuscipes* species*.* Apparent density (AD) ranged from 0.58 to 3.67 tsetse flies per trap per day for all the villages. However, the average FTD in the two districts did not differ significantly (Mann-Whitney U test, U = 10.0, *p* = 0.602). The average age of flies caught in Adjumani district was 30 days (Mean Wing Fray Value, MWFV = 3.8 ± 0.298) while for Moyo district was 25 days old (MWFV = 3.3 ± 0.253) (Table 2).

**Table 2:** Results of entomological surveys in Adjumani and Moyo districts in Northwest Uganda

Village/site	X	Y	No of traps	Male	Female	Catch (M ± SE)	FTD	MWFV	Estimated age
Olwi	3.149863	31.681045	10	10	18	2.7 ± 1.08	0.93	3.8	30 days
Osugo East	3.200765	31.722688	10	32	49	7.9 ± 0.81	2.7	2.8	21 days
Olobo	3.384375	32.011374	12	55	60	9.6 ± 2.29	3.19	4.3	34 days
Oringya	3.486135	32.010335	11	14	21	3.2 ± 1.16	1.06	4.5	36 days
Pagirinya	3.377809	31.994145	10	29	32	6.1 ± 1.43	2.03	3.6	28 days
Ori	3.64733	31.78997	10	10	29	3.9 ± 0.86	1.3	4.2	33 days
Orubakulem	3.691983	31.780385	13	64	79	11.9 ± 2.80	3.67	3	23 days
Lea	3.592517	31.606833	11	30	49	7.1 ± 1.52	2.39	2.8	21 days
Cefo	3.6055	31.651733	10	12	8	3.9 ± 0.86	0.67	3.5	27 days
Moyipi	3.63794	31.71825	12	13	8	2.1 ± 0.62	0.58	3	23 days
			**109**	**269**	**353**				

MWFV refers to Mean Wing Fray Value; X and Y refer to the values of X and Y coordinates of the villages

### Molecular identification of trypanosomes species and sub-species

**Fig. 3:**
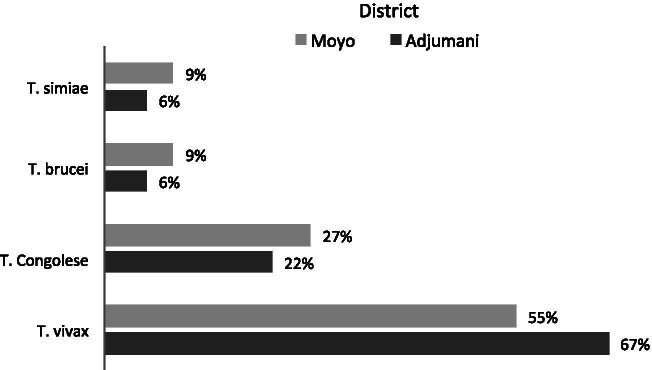
Overall prevalence of the different trypanosome species and subspecies in Moyo and Adjumani Districts, Northwestern Uganda

We analyzed 44% (272/622) of the collected tsetse samples for detection of trypanosomes. Out of the 29 infected tsetse flies found in both districts, *Trypanosoma vivax* was the most prevalent species accounting for 6.62% of the infections, followed by *T. congolense* (all three sub types Kilifi, Forest and Savanna; 2.57%, and *T. brucei* and *T. simiae* each at 0.74% (Table 3). The results of investigation for *T. brucei gambiense* turned out negative, and since we did not expect the other *Trypanozoon* (*T. evansi* and *T. b rhodesiense*) to be present in this geographical area, we concluded that the subspecies was *T. brucei brucei*. There were two cases of mixed infections with *T. brucei brucei/T. vivax*. The overall prevalence of the different species and sub-species in the two districts is shown in Fig. 3.

**Table 3:** Rate of tsetse trypanosome infection in the villages of Moyo and Adjumani districts, northwestern Uganda

District	Village	Total flies examined	Total infected	Infection rate (%)	***T. vivax***	***T. brucei***	***T. simiae***	***T. Congolense***
Adjumani	Olwi	12	0	0	0	0	0	0
	Osugo East	35	4	11.42	3	0	0	1
	Olobo	60	11	18.33	7	1	1	2
	Oringya	14	2	14.2	1	0	0	1
	Pagirinya	29	1	3.45	1	0	0	0
Moyo	Ori	16	1	6.25	1	0	0	0
	Orubakulem	60	7	11.67	3	1	1	2
	Lea	30	3	10	2	0	0	1
	Cefo	8	0	0	0	0	0	0
	Moyipi	8	0	0	0	0	0	0
Total		272	29	10.67	18 (6.62%)	2(0.73%)	2(0.73%)	7(2.57%)

### Prevalence of trypanosome infection

The overall infection rate was 10.67% (29/272). The prevalence of trypanosome infection was not significantly associated with the fly’s sex (GLM = χ^2^ = 1.723, *P* = 0.189, df = 1, *n* = 272) and district of origin (χ^2^ = 0.018, *P* = 0.895, df = 1, n = 272). However, trypanosome infection was significantly associated with the fly’s age based on wing fray category (χ^2^ = 22.374, *P* < 0.001, df = 1, n = 272) and the prevalence of infection was higher among the very old than the young tsetse. In Adjumani district, the village with the highest prevalence of infection was Olobo (18.3%), followed by Oringya (14.2%), Osugo East (11.4%) and Pagirinya (3.45%). In Moyo District, the highest rates of infection were recorded in Orubakulem (11.67%), followed by Lea (10%) and then Ori (6.25%). There was no infected fly in Cefo and Moyipi villages (Table 3).

### Blood meal identification

**Fig. 4:**
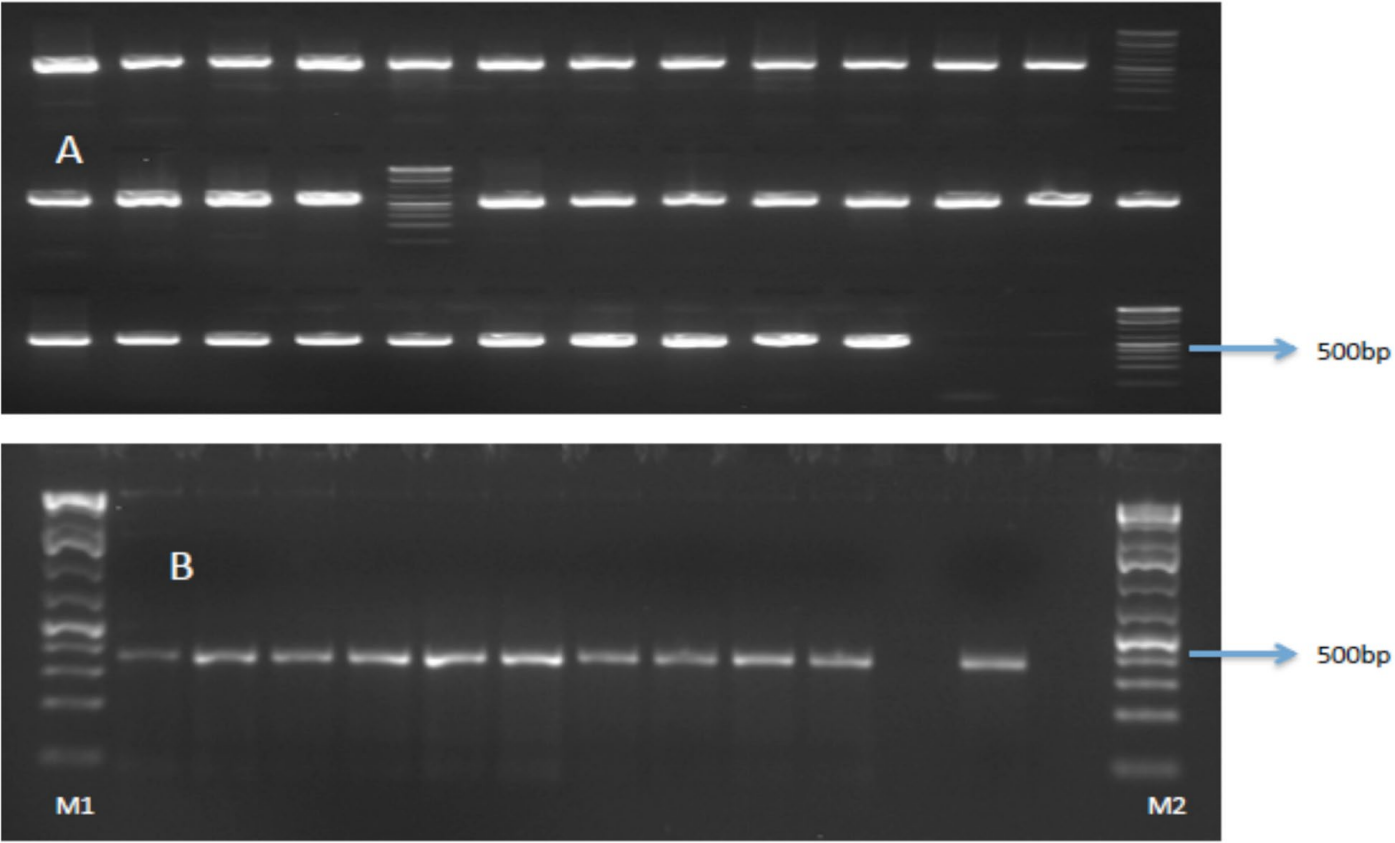
Representative gel photograph showing 650 bp (**A**) and 359 bp (**B**) fragments size of COI and cyt b gene amplification products. Lanes M1, M2: 75 bp marker

A total of 34 blood-engorged flies’ midgut samples were analyzed to determine the sources of the blood meal. Amplification of the COI and *Cyt b* genes all gave a 650 bp and 359 bp PCR products, respectively, on 1.8% agarose gels (Fig. 4).

Hosts were identified in 94.1% (32/34) of analyzed samples, with sequence identities ranging from 96 to 100% in identity with reference sequences in Genbank. Tsetse hosts identified included cattle (19/32) (GenBank accession numbers: OK639114, OK639115, OL310103, OL310104, OL310709, OL310710, OL310819, OL310820, OL310821, OL310898, OL310909, OL310911, OL314660, OL314661, OL314664, OL314665, OL321945, OL322022 and OL322028), Humans (5/32) (GenBank accession numbers: OL310817, OL310818, OL310912, OL310900 and OL321877), Nile monitor lizard (5/32) (GenBank accession numbers: OL310899, OL310901, OL310902, OL310910 and OL321790), African mud turtle (2/30) (GenBank accession number: OL314662 and OL318407), and the African Savanna elephant (1/30) (GenBank accession number: OL310913) (Table 4).

**Table 4:** Details of identification and accession numbers for all reference sequences for COI and cytb gene sequences generated

Host species identified	N (***n*** = 32)	Genbank ID (COI)	Closest match on BLAST - Genbank ID (Cyt b)
*Humans (Homo sapiens)*	05	OL310817, OL310818, OL310912, OL310900, OL321877	AY509658 GU123040.1 HM036565 GU123040.1
*Varanus niloticus*	05	OL310899, OL310901, OL310902, OL310910, OL321790	ND
*Cattle (Bos taurus)*	19	OK639114, OK639115, OL310103, OL310104, OL310709, OL310710, OL310819, OL310820, OL310821, OL310898, OL310909, OL310911, OL314660, OL314661, OL314664, OL314665, OL321945, OL322022 OL322028	ND
*Pelusios chapini*	02	OL314662, OL318407	ND
*African Savanah Elephant (Loxodonta africana*)	01	OL310913	ND

Data combine both cytb and COI, and GenBank accession numbers for all CO1 sequences given, and where not determined indicated as ND

## Discussion

This study assessed the apparent density and infection rates of tsetse flies in Adjumani and Moyo Districts in northwestern Uganda, a known African trypanosomiasis endemic focus. The only species of tsetse caught was *Glossina fuscipes fuscipes,* probably due to the nature of vegetation and our sampling scheme that mainly targeted the edges of major rivers and streams as well as peri-domestic environments. *Glossina fuscipes fuscipes* is known to generally disperse along waterways, following either riverbeds or the edges of gallery forests, where they are able to survive low humidity conditions during dry seasons [31–33].

Female flies were consistently higher than males in most traps in both districts. This finding is similar to results of a survey in Mukono district, southeastern Uganda where the percentage of females was higher than that of males in all study months [34]. Females are generally known to live longer than males [35] hence their ususally higher abundnace in a typical population. Additionally, female tsetse are also known to be more prolific feeders; male flies feed every 3–4 days [36] whereas females take blood meals regularly due to their role in reproduction [37], which increases their chances of getting trapped as they seek for blood meals.

In the present work, we found no significant difference in apparent density between the two districts implying that the similarity in biotopes present favorable habitats for tsetse in both districts. The average AD obtained in this study is similar to the findings by Azabo et al. [38] in mid northern Uganda. However, it’s lower than those reported in southeastern Uganda by Waiswa et al. [39]. This observed difference could be attributed to variations in the environmental and agro-ecological conditions and the season in the study areas. Northern and northwestern Uganda differ significantly in climate, especially in annual precipitation, from the southern part of the country [40]. Southern Uganda has a somewhat cooler climate and is more humid, with mean annual rainfall near Lake Victoria often exceeding 2100–3000 mm; the high temperature varies by 2–3 °C over the year, with a mean daily high being around 26 °C. In the north and northwest, the rainfall is between 1000 and 2000 mm, and temperature varies by 5 °C over the year, with the mean daily high being 29 °C [41]. The presence of unique climatic background creates different tsetse population dynamics, and may be responsible for the observed difference in AD.

Our results showed that 10.7% of tsetse flies in both Adjumani and Moyo Districts were infected by at least one trypanosome species. This figure is relatively high given that infection rates are known to be generally low in tsetse flies [42]. Other studies in Uganda have reported lower rates; Azabo et al. [38] got an infection rate of 5.6% in tsetse flies from mid northern Uganda districts of Lira, Apac and Kole. Waiswa et al. [43] got an even lower rate (1.55%) in southeastern Uganda, although their study was based on microscopic examination only. However, the infection rate reported in this study is lower than those reported from other studies in Sub-Saharan Africa such as Simo et al. [44] who reported 19.3% at the Malanga sleeping sickness foci in the Democratic Republic of Congo and Simo et al. [45] who reported 25.5% in Cameroon. Our comparatively higher infection rates than reported elsewhere in Uganda can be explained by the fact that livestock owners in Adjumani and Moyo rarely use trypanocidal drugs due to its high costs (District Veterinary Officer, Adjumani, Personal Communication, July, 2018).

Our results indicated that sex of fly had no significant association with trypanosome infection. This contrast with the suggestion that fly sex appears to influence susceptibility to trypanosome infection with females being more susceptible to parasite infection than their male counterparts [46–49]. Females are known to live longer than males [35], and infected female tsetse that live longer could increase the number of infected females compared to male flies. However, there have been instances where higher number of infections were associated with male flies [46, 47, 49]. Thus, the precise associations of trypanosome infection with sex remain unresolved. Furthermore, prevalence of trypanosome infection correlated significantly with age, with older flies having a higher level of infection than their younger counterparts. This is consistent with the observation that some trypanosomes require a longer time for maturation for example, *T. congolense* at 24 °C takes up to 15–20 days [31] hence older flies expected to have more infections.

The most common trypanosome species in tsetse flies in the two districts was *T. vivax*, accounting for 67% of the infected flies. Magona et al. [50] found similar results in studies of *Glossina fuscipes fuscipes* tsetse flies in Southeastern Uganda. In this study, our traps caught only *Glossina fuscipes fuscipes*, which known to be more susceptible to *T. vivax* infections [51]. *Trypanosoma vivax* is also known to have a shorter developmental life cycle and can be mechanically transmitted by haematopahgous insects, which may explain its predominance in the surveyed samples [52]. An earlier study by Angwech et al. [53] in Amuru and Nwoya districts, which border the study area in the present study, found that *T. vivax* was also the most predominant trypanosome species in cattle. Our results are also consistent with several other previous studies on tsetse and trypanosomes ecology in Uganda [39, 43, 54].

Our analyses did not find human infective trypanosome species in the tsetse samples analysed. A recent epidemiological survey in the neighbouring Arua and Koboko districts, all of which are within the same gambiense focus, also reported no cases of sleeping sickness in the region [14]. It is possible therefore that the transmission of *T. b. gambiense* has been decisively suppressed and prevalence reduced to near elimination. Active case detection through mass community screening by the Ugandan National Sleeping Sickness Programme and Medecins Sans Frontières (MSF) France between 1987 and 2002 [55], and by MSF Spain and the Ministry of Health (MoH) supported by WHO in 2010 and 2011 [13], plus associated vector control activities in the region, was probably responsible for this decline. With regards to vector control, however, a lull in activities due to lack of funding and other logistical challenges [56] has probably led to an increase in tsetse numbers in the region as portrayed by the high vector density in most sites in this study. Lately though, vector control using tiny targets have been initiated with encouraging results in terms of a reduction in tsetse numbers [55]. The absence of detection of *T. b. gambiense*, however, cannot be conclusive due to our relatively small sample size. We encourage more extensive screening of the vectors since it is generally known that prevalence of gambiense HAT disease amongst wild tsetse populations is often extremely low [57–59].

In the present study, we used two genetic markers (COI and cytb) for identification of tsetse blood meal sources in Adjumani and Moyo. The COI gene has a higher taxonomic coverage in reference databases [60] but *Cyt b* has been shown to be better than COI at detection of the origin of digested blood meals in some insect vectors [61]. Muturi et al. [26] consistently failed to amplify tsetse vertebrate host DNA from *Glossina swynnertoni* using the COI gene for flies caught in the Serengeti. However, by complementing the analyses of the COI gene with the analyses of *Cyt b*, we were able to identify blood meal sources of the caught tsetse. Conversely, in the study of Petterson et al. [62], the authors used COI for blood meal analyses for samples that did not produce results with *Cyt b* primers in order to increase the opportunity for host identification. Therefore, where possible, the use of both markers for detection of blood meal sources in vertebrates is a logical application since the COI and *Cyt b* DNA barcoding regions from more species are being sequenced and submitted to public databases.

Sequence analyses showed various blood meal sources, with cattle, humans and monitor lizards being the predominant hosts. *Glossina fuscipes fuscipes* is known to be a non-preferential feeder, with blood meals taken from the most available hosts [63]. However, in Adjumani and Moyo districts, tsetse mostly feed on humans and cattle due to the fact that the environment is highly anthropized and wild animals are rare, except in Zoka forest, which has abundant wildlife [64], an area we did not sample. Another probable reason for the high cases of blood meal from cattle, and to some extent humans, is that this survey was done in the dry season when it is common that animals, especially cattle, are taken to the banks of rivers and streams to graze and drink water. As such, *Glossina fuscipes fuscipes*, a riverine species, gain access to the cattle and humans who attend to the animals and utilize the water for domestic and other uses. There was, however, one curious case of a blood meal taken from an elephant. This particular fly was trapped from Arinyapi sub-county in Adjumani district, which borders southern Sudan. Trans-boundary elephants especially from Nimule National Park in South Sudan that have on several occasions destroyed numerous acres of food crops belonging to hundreds of small-scale farmers in the two sub-counties of Arinyapi and Dzaipi [65] are seemingly potential hosts of *Glossina* tsetse in the study area. Results of blood meal analyses furthermore highlight the importance of reptiles, especially monitor lizards, as important hosts for *Glossina fuscipes fuscipes* tsetse. Previous studies in southeastern Uganda [43, 63, 66] and on the shores of Lake Victoria in Kenya [67] identified monitor lizards as an important food source for tsetse. It has been proposed that the preference for reptiles may simply reflect an ecological ‘concordance’ between flies and lizards; both are cold-blooded and probably with similar diurnal pattern in the environment where they co-exist [67]. Reptiles are even suspected to be natural cryptic reservoirs of human infective trypanosomes [68].

## Conclusion

The present study has shown an infection rate of 10.8%, with all infections attributed to trypanosome species that are causative agents of the animal disease only. However, the absence of detection of human infective trypanosomes in tsetse flies in this endemic focus require verification using large-scale passive and active seasonal entomological and human screening to ascertain if HAT is not a public health problem anymore in the study area. Finally, this study reveals that cattle, humans and reptiles are among the most important blood meal sources for tsetse flies in the region and should be considered in the design of tsetse and trypanosomiasis control interventions.

### Study limitations

We acknowledge the following limitations:

- The small sampling size for detection of trypanosome species and sub-species involving only 272 tsetse out of the 622 sampled and blood meal analyses on only 34 blood-engorged adult tsetse.
- The tsetse aging techniques used – wing fray analyses- was not the most accurate as opposed to the ovarian aging approach.

## Data Availability

The datasets generated and/or analysed during the current study are available in Genbank with the following accession numbers: *Bos taurus* (cattle): OK639114, OK639115, OL310103, OL310104, OL310709, OL310710, OL310819, OL310820, OL310821, OL310898, OL310909, OL310911, OL314660, OL314661, OL314664, OL314665, OL321945, OL322022 and OL322028; *Homo sapiens* (humans): OL310817, OL310818, OL310912, OL310900 and OL321877; *Varanus niloticus* (Nile monitor lizard): OL310899, OL310901, OL310902, OL310910 and OL321790; *Pelusios chapini* (African mud turtle): OL314662 and OL318407, and *Loxodonta africana* (African Savanna elephant): OL310913.

## Abbreviations

AD: Apparent Density
COI: Cytochrome Oxidase I
FAO: Food and Agricultural Organization
HAT: Human African Trypanosomiasis
AAT: Animal African Trypanosomiasis
OD: Optical Density
PCR: Polymerase Chain Reaction
TgsGP: *Trypanosoma gambiense* specific Glycoprotein
WHO: World Health Organization
